# Patterns in the Parathyroid Response to Sodium Bicarbonate Infusion Test in Healthy Volunteers

**DOI:** 10.1155/2014/704394

**Published:** 2014-04-08

**Authors:** Theodossis S. Papavramidis, Olympia E. Anastasiou, Ioannis Pliakos, Nick Michalopoulos, Mike Polyzonis, Konstantina Triantafyllopoulou, Georgia Kokaraki, Spiros Papavramidis

**Affiliations:** ^1^3rd Department of Surgery, AHEPA University Hospital, Aristotle University of Thessaloniki, Thessaloniki, 54655 Macedonia, Greece; ^2^Department of Endocrinology and Metabolism, AHEPA University Hospital, Aristotle University of Thessaloniki, Thessaloniki, 54655 Macedonia, Greece; ^3^Department of Immunology, AHEPA University Hospital, Aristotle University of Thessaloniki, Thessaloniki, 54655 Macedonia, Greece

## Abstract

*Background*. The sodium bicarbonate infusion test evaluates the function of the parathyroid glands. The present study aims to evaluate the range of parathyroid response in healthy individuals and the potential influence of various factors. *Methods*. Fifty healthy volunteers were subjected to the test. Levels of vitamin D, calcium, albumin, and PTH were measured before infusion. PTH was measured at 3, 5, 10, 30, and 60 minutes after infusion. *Results*. A curve describing the response of parathyroids to the test was drawn. Twenty percent of the subjects had blunted PTH response. No significant difference was observed between normal and blunted responders concerning age, BMI, baseline PTH, or calcium levels. Nonetheless, there was a significant difference in vitamin D levels (*P* = 0.024). *Interpretation*. The test is easy to perform and may be used for everyday screening. It has to be clarified whether our observations are, at least partly, produced due to the presence of individuals with a constitutively blunted response or if low levels of vitamin D decrease the ability of the parathyroids to respond. Whichever the case, PTH response of normal individuals to sodium bicarbonate infusion test is more varied than previously thought and vitamin D levels influence it.

## 1. Introduction

The parathyroid glands are small, brownish-tan glands located in the space around the thyroid gland [1]. They play a central role in regulating serum levels of calcium through a complex feedback loop involving various factors such as parathormone (PTH), serum ionized calcium levels, and vitamin D. The key organs involved in this process include the parathyroid glands, gastrointestinal tract, kidneys, and skin [2]. It is easily comprehensible that normal calcium homeostasis plays a major role since it influences cellular function throughout the body.

In order to evaluate the normal function of the parathyroids several biochemical markers, such as serum calcium, phosphorus, and PTH, are employed. However, these markers are not dynamic but reflect the instant good functioning of the gland. On the other hand, the number of available provocative tests for the evaluation of parathyroid glands function is limited. Ethylenediaminetetraacetic acid (EDTA) infusion test and sodium citrate infusion test have been used in the past to evaluate parathyroid gland function [3–5], while Iwasaki et al. [6] have introduced a new test using sodium bicarbonate infusion.

The aim of the present study was to evaluate the parathyroid glands response in healthy individuals, using the sodium bicarbonate infusion test. Moreover, we assessed the potential influence various factors such as age, body mass index (BMI), vitamin D, calcium, albumin, phosphate, and magnesium on the provoked PTH secretion.

## 2. Material and Methods

**Figure 1:**
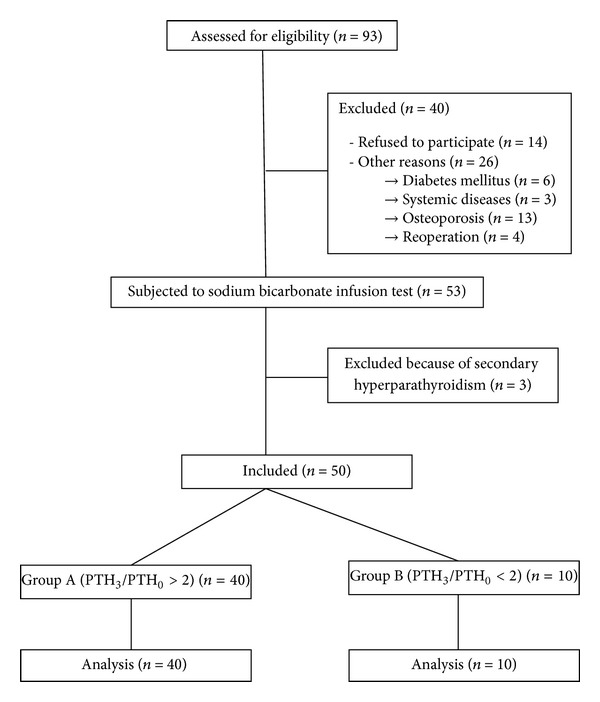
Flow diagram of the participants entering the study.

Ninety-three volunteers were assessed for eligibility in the study. The inclusion criteria were (i) age more than 18 years and (ii) acceptance to participate to the study (signed informed consent form). The exclusion criteria were (i) previous operation at the thyroid, parathyroid or neck irradiation, (ii) participation in another clinical trial which may affect this study's outcome, (iii) noneuthyroid condition, (iv) primary or secondary hyperparathyroidism, (v) primary or secondary hypoparathyroidism, (vi) diabetes mellitus, (vii) chronic renal failure, (viii) systemic diseases (e.g., infections, neoplasms), (ix) hypoalbuminemia, (x) osteoporosis and use of drugs that influence calcium metabolism (vitamin D analogues, oral calcium supplements, biphosphonates, teriparatide, thiazide diuretics, etc.), and (xi) osteomalacia. The flow diagram of the participants is presented in Figure 1.

According to the abovementioned criteria 50 individuals were eligible to participate. In the following, the sodium bicarbonate infusion test results the subjects were divided into two groups according to the fold increase of PTH at 3 min (PTH at 3 min/PTH initial). Group A consisted of subjects with more than twofold increase, while group B included subjects with less than twofold increase. The abovementioned study adhered to the tenets of the declaration of Helsinki as revised in 1989 and was approved by the Ethics Committee of AHEPA University Hospital (Aristotle University of Thessaloniki).

### 2.1. Study Protocol

Serum levels of total calcium, albumin, 25-OH vitamin D (by radioimmunoassay, Diasorin, MN, USA), and PTH were measured before infusion. The sodium bicarbonate test was performed as previously described [6, 7]. The volunteers were in fasting state for at least 4 hours and 35 mL/m^2^ of body surface of 8.4% sodium bicarbonate solution was infused in one arm over 2 minutes and blood samples for the determination of serum PTH were drawn before (PTH_0_) and at 3 (PTH_3 min_), 5 (PTH_5 min_), 10 (PTH_10 min_), 30 (PTH_30 min_), and 60 minutes (PTH_60 min_) after the infusion of the other. Infusion of sodium bicarbonate causes acute metabolic alkalosis and in turn increases the binding of calcium to albumin leading to a decrease of the ionized calcium in blood [7]. No side effects were observed during the sodium bicarbonate infusion test except for a transient lips or/and hands paresthesia, metallic taste, and slight pain in the arm. However all these symptoms were transient and lasted about five minutes from the beginning of the infusion.

### 2.2. Statistical Analysis

SPSS 17.0 for Windows (SPSS Inc., Chicago, IL) was used for statistical analysis of the data; *t* tests, nonparametric tests (Mann-Whitney *U*), and repeated measures ANOVA were used to compare groups as applicable. GraphPad Prism 4 (GraphPad Software, Inc., La Jolla, CA) was used to measure the area under the curve (AUC). Data are presented as mean values ± SEM. Differences were considered to be significant if *P* < 0.05. Normality was assessed with Shapiro-Wilk test. PTH levels did not follow normal distribution, so their square roots were used to perform ANOVA. The PTH_3 min_/PTH_0_ and 25(OH)D did not follow normal distribution, so their logarithms were used to perform Pearson's correlation test. Concerning the *t* tests, all were two-tailed, and according to the distribution of each tested variable, a parametric or a nonparametric test was used.

## 3. Results

**Figure 2:**
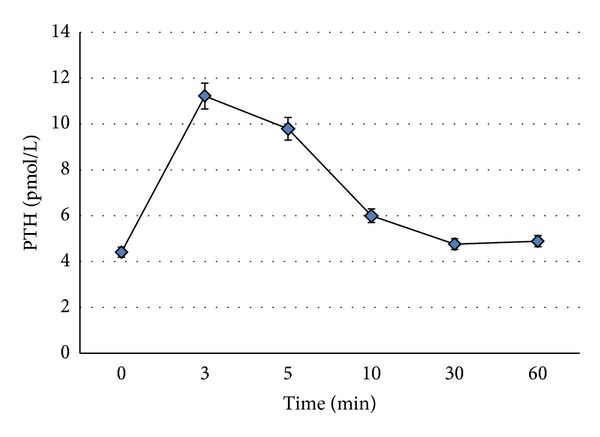
PTH levels after sodium bicarbonate infusion. There were statistically significant differences to PTH levels after infusion compared to baseline PTH levels.

The epidemiologic characteristics of all participants, as well as the various parameters measured (PTH values at various moments, total calcium, and vitamin D levels) are presented in Table 1. Concerning PTH measurements in the total population after sodium bicarbonate infusion, we observed that PTH levels rise and remain elevated at 3 (*P* < 0.001), 5 (*P* < 0.001), and 10 minutes (*P* < 0.001) (Figure 2). No statistical significant difference was observed between the baseline values and PTH_30 min_, while a significant difference was observed between baselines PTH and PTH_60 min_ (Figure 2, Table 1). The mean ratio PTH_3 min_/PTH_0_ was 2.57 (ranging from 1.4 to 4.67).

Based on the PTH_3 min_/PTH_0_ ratio, the participants were divided into two groups. Group A (*n* = 40) consisted of participants with PTH_3 min_/PTH_0_ ratio >2, while group B (*n* = 10) consisted of participants with ratio <2. In order to explain this difference of response between the two groups age, BMI, baseline levels of calcium, albumin, phosphate, and magnesium were compared. No statistically significant differences were observed (Table 1). The only parameter presenting statistically significant difference was vitamin D (*P* = 0.024). A positive correlation was observed between the logarithm of 25(OH)D and the logarithm of PTH_3 min_/PTH_0_ (*r* = 0.304, *P* = 0.034).

**Figure 3:**
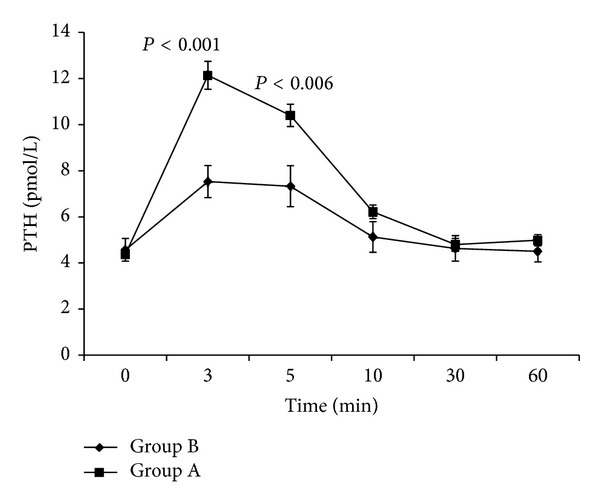
PTH levels after sodium bicarbonate infusion. There was a statistically significant difference between the two groups at 3 and 5 minutes.

As far as it concerns the comparison of the PTH secretion curves between the two groups, we observe that at 3 min after the infusion, PTH values for both groups rise from baseline secretion to a maximal peak and then slowly decrease and return to baseline values at 30 min (Figure 3). Statistical significant differences between the PTH of the two groups is observed only for PTH_3 min_ (*P* < 0.001) and PTH_5 min_ (*P* = 0.006). Despite the differences concerning the peak secretions, the total quantity of PTH secreted during the 60 minutes after the infusion was not statistically significant (*P* = 0.061) (Table 1).

## 4. Discussion

The dynamic monitoring of the parathyroid glands is very important since calcium homeostasis affects cellular functioning throughout the body. Moreover, it is not always evident which is the best way to monitor postoperative function of the gland [8]. The number of available provocative tests for the evaluation of the parathyroid function is limited to EDTA infusion test, sodium citrate infusion test, and, recently, sodium bicarbonate infusion test [3–7]. We have chosen to employ this last test for a number of reasons. The EDTA infusion test is time-consuming and has adverse cardiovascular effects making it an unlikely candidate for parathyroid gland function evaluation in clinical practice [6]. The sodium citrate infusion test has been used to outline the full spectrum of the relationship between serum ionized calcium and PTH but is lengthy and difficult to apply in large scale [5]. We found sodium bicarbonate infusion test to be a safe, fast, and easy alternative, which would make it ideal for the evaluation of parathyroid function in a clinical setting.

In the study which introduced the sodium bicarbonate infusion test, Iwasaki et al. observed that normal subjects have an average 4-fold increase of PTH levels at 3 min after the infusion, while patients suffering from hypoparathyroidism or hyperparathyroidism have a blunted response of lower than twofold increase [6]. We observed that the PTH_3 min_/PTH_0_ in our subjects was lower than previously described. A 20% of our group presented a lower than expected increase of PTH levels, if one accepts the twofold increase as the limit of totally normal parathyroid function. Possible reasons for the observed differences are the fact that our sample was larger and belonged to a different population than this of the previous study. Furthermore, the mean age of our group was greater than this of Iwasaki et al. As a final point, in our study 46% of our subjects had 25(OH)D levels lower than 20 ng/mL. This is in accordance to previous studies performed in the Mediterranean region and in Greece in particular, showing low levels of vitamin D [9–11]. Possible reasons for the high prevalence of vitamin D deficiency include a more pigmented skin, lower dietary intake of vitamin D, decreased use of dietary supplements, and avoidance of sunlight exposure compared to inhabitants of Northern Europe and North America [12].

As previously noted, 20% of the volunteers present a PTH_3 min_/PTH_0_ ratio less than 2. In order to identify possible factors leading to this impaired parathyroid function, we created two groups (A and B) and compared several factors which influence baseline PTH levels and could contribute to altered response of PTH secretion. These factors included age, BMI, serum calcium, phosphate, magnesium, and 25(OH)D levels [13–19]. Both our groups were comparable concerning age and BMI, while calcium, albumin, phosphate, and magnesium levels were within normal range as well and did not differ significantly between the groups. In contrast, 25(OH)D levels differed significantly between the groups. Group A had higher 25(OH)D levels than Group B. Furthermore vitamin D levels correlated positively with the increase of PTH_3 min_. Similarly to our study a previous published study, employing EDTA infusion test, reported that basal vitamin D status appeared to be a determinant of the degree of the PTH response in black but not white women with the peak PTH level being inversely correlated with levels of 25OHD [20]. Indeed in group B the mean of 25(OH)D level fell below normal levels, with most of the individuals suffering from mild vitamin D insufficiency and one individual from moderate. Interestingly this individual had, in addition, the most blunted response. In contrast, the mean of 25(OH)D level of Group A was above normal levels.

The data of the present study come from healthy volunteers leaving the field of everyday methodological practice free to be developed and explored. On a clinical basis, recently Annerbo et al. published that there is a left-shifted relation between calcium and PTH in Graves' disease [21], leading to increased postoperative hypocalcemia in these patients. May be the sodium perfusion test could help identify the Graves patients that are more susceptible to develop postoperative hypocalcemia. Concerning parathyroid surgery, the existence of high and low responders, as described in this study, may lead to confusing results when performing a parathyroidectomy under general anesthesia. It is known that anesthetic technique influence PTH levels during the minutes following induction to anaesthesia [22], in that way excising a possible parathyroid adenoma too fast in a high responder, may lead to a smaller fall of PTH than expected. The possible clinical applications of the data presented in the present paper, however, are to be explored in future trials.

The sodium bicarbonate infusion test is easy to perform and may be used for screening of the parathyroid function in everyday clinical practice. The range of responses in our group is shifted to the left compared to the initial results reported by Iwasaki et al. It is a matter of discussion and further investigation to clarify whether our observations are, at least partly, produced due to the presence of individuals with a constitutively blunted response of PTH or if low levels of vitamin D decrease the ability of the parathyroid glands to respond to a potent stimulus such as acute hypocalcemia. Whichever the case, it is shown that PTH response of normal individuals to sodium bicarbonate infusion test is more varied than previously thought and vitamin D levels influence it.

## Figures and Tables

**Table 1:** The various PTH values, total calcium and vitamin D levels of all the patients.

	Total *n* = 50	Group A *n* = 40	Group B *n* = 10	*P* (A versus B)
Age (y) [SD]	47.58 [2.05]	48.42 [2.12]	44.40 [5.81]	0.432
BMI (Kg/m^2^) [SD]	26.15 [0.58]	26.69 [0.63]	24.00 [1.27]	0.065
25(OH)D (ng/mL) [SD]	24.16 [2.11]	26.09 [2.46]	15.60 [1.92]	0.024*
Calcium (mg/dL) [SD]	8.86 [0.08]	8.85 [0.10]	8.87 [0.17]	0.895
Albumin (g/dL) [SD]	4.35 [0.06]	4.35 [0.67]	4.32 [0.15]	0.809
Phosphate (mg/dL) [SD]	3.43 [0.08]	3.37 [0.10]	3.60 [0.16]	0.257
Magnesium (mg/dL) [SD]	2.12 [0.04]	2.13 [0.05]	2.10 [0.06]	0.914
PTH at 0 min (pmol/L) [SD]	4.41 [0.17]	4.37 [0.17]	4.57 [0.50]	0.639
PTH at 3 min (pmol/L) [SD]	11.22 [0.57]	12.14 [0.61]	7.53 [0.70]	<00.1*
PTH at 5 min (pmol/L) [SD]	9.79 [0.45]	10.40 [0.48]	7.33 [0.89]	0.006*
PTH at 10 min (pmol/L) [SD]	6.00 [0.28]	6.22 [0.30]	5.13 [0.66]	0.117
PTH at 30 min (pmol/L) [SD]	4.76 [0.24]	4.80 [0.27]	4.63 [0.55]	0.747
PTH at 60 min (pmol/L) [SD]	4.89 [0.21]	4.98 [0.24]	4.50 [0.16]	0.367

Total PTH secretion (pmol/L) [SD]	331.17 [15.65]	345.78 [15.24]	272.73 [46.52]	0.061

*When *P* statistically significant.
